# British Pastry

**Published:** 1883-11

**Authors:** 


### BRITISH PASTRY.

Is there something in the climate of this country or the mental and physical attributes of the people which prevents the creation of palatable pastry? The Royal Society might entertain this question with probably far more profit to the pleasure of the people of Great Britain than has been derived from a hundred of its philosophic investigations. Years roll on, and we do make progress in some matters, albeit the heavy drag-chains of those British Bourbons who learn nothing and forget nothing hold us back; but the boggy bun and leathery tart torily hold their own, and the palate of maturity recoils with loathing from these horrid cakes. How different it is in some—indeed, in most—other countries! Cross the Channel, and the man who here refuses even to look at the pastry-cook's counters is found in Brussels or Paris devouring tartlets and "goodies" like a schoolboy. In Switzerland, too, pastry is delicious. Indeed, in that gastronomic *erebus* (as the *gourmet* Ford bitterly dubbed Spain), the Engadine, it is, or was, almost the only edible article save veal. Why do not some of these Engadiners emigrate to English towns? They go to great Continental and American cities. From the neighboring Canton of Ticino went forth Delmonico, as the apostle of gastronomy to Columbia; and when you once begin eating his tartlets it is hard to stop, despite their prohibitory prices. Possibly the dampness of our climate may have a "soggy" influence on pastry here. Whatever the cause, there is no doubt that we bear away the bell for badness. Where to get in London even such tarts as may be got in half a dozen shops in Brussels or Paris I could not tell my dearest friend.—*London World.*

---

Reproof and Rejoinder.—An Elder of the Kirk having found a little boy and his sister playing marbles on Sunday, put his reproof in this form, not a judicious one for a child: "Boy, do you know where children go to who play marbles on the Sabbath day?" "Ay," said the boy, "they gang down to the field by the water below the brig." "No," roared out the Elder, "they go to hell and are burned." The little fellow, really shocked, called to his sister, "Come awa, Jeanie, here's a man swearing."

---

An elderly lady in Athens, Ga., owns the original manuscript of "Home, Sweet Home," as written by John Howard Payne. The words of the poem as first written are all interlined, with here and there an endearing expression from the writer to the lady who now holds it. In the old days, Payne was devotedly attached to her, and she has many of his letters. She has been offered a large sum for the manuscript.

